# A comprehensive artificial intelligence framework for dental diagnosis and charting

**DOI:** 10.1186/s12903-022-02514-6

**Published:** 2022-11-09

**Authors:** Tanjida Kabir, Chun-Teh Lee, Luyao Chen, Xiaoqian Jiang, Shayan Shams

**Affiliations:** 1 The University of Texas Health Science Center at Houston, School of Biomedical Informatics, Houston, TX USA; 2 Department of Periodontics and Dental Hygiene, The University of Texas Health Science Center at Houston School of Dentistry, Houston, TX USA; 3 Department of Applied Data Science, San Jose State University, One Washington Sq, San Jose, CA 95192 USA

**Keywords:** Deep learning/machine learning, Dental informatics/bioinformatics, Computer vision, Radiography, Imaging

## Abstract

**Background:**

The aim of this study was to develop artificial intelligence (AI) guided framework to recognize tooth numbers in panoramic and intraoral radiographs (periapical and bitewing) without prior domain knowledge and arrange the intraoral radiographs into a full mouth series (FMS) arrangement template. This model can be integrated with different diseases diagnosis models, such as periodontitis or caries, to facilitate clinical examinations and diagnoses.

**Methods:**

The framework utilized image segmentation models to generate the masks of bone area, tooth, and cementoenamel junction (CEJ) lines from intraoral radiographs. These masks were used to detect and extract teeth bounding boxes utilizing several image analysis methods. Then, individual teeth were matched with a patient’s panoramic images (if available) or tooth repositories for assigning tooth numbers using the multi-scale matching strategy. This framework was tested on 1240 intraoral radiographs different from the training and internal validation cohort to avoid data snooping. Besides, a web interface was designed to generate a report for different dental abnormalities with tooth numbers to evaluate this framework’s practicality in clinical settings.

**Results:**

The proposed method achieved the following precision and recall via panoramic view: 0.96 and 0.96 (via panoramic view) and 0.87 and 0.87 (via repository match) by handling tooth shape variation and outperforming other state-of-the-art methods. Additionally, the proposed framework could accurately arrange a set of intraoral radiographs into an FMS arrangement template based on positions and tooth numbers with an accuracy of 95% for periapical images and 90% for bitewing images. The accuracy of this framework was also 94% in the images with missing teeth and 89% with restorations.

**Conclusions:**

The proposed tooth numbering model is robust and self-contained and can also be integrated with other dental diagnosis modules, such as alveolar bone assessment and caries detection. This artificial intelligence-based tooth detection and tooth number assignment in dental radiographs will help dentists with enhanced communication, documentation, and treatment planning accurately. In addition, the proposed framework can correctly specify detailed diagnostic information associated with a single tooth without human intervention.

**Supplementary Information:**

The online version contains supplementary material available at 10.1186/s12903-022-02514-6.

## Background

Tooth detection and tooth number assignment in radiographs are essential for clinical record-keeping [1], dental abnormality diagnosis [2–4], surgical and orthodontic planning [5], reducing the workload of human experts [6], easy charting, and communication among dental professionals [7]. Usually, two types of dental radiographs are used for clinical diagnosis: (i) extraoral- cone-beam computed tomography (CBCT) and panoramic, (ii) intraoral- periapical and bitewing. CBCT images are used to examine underlying teeth, bone structure, and nerve pathways in three dimensions. Panoramic, periapical, and bitewing images are two-dimensional and more commonly available than CBCT. Panoramic radiographs include the full view of the mouth, capturing all teeth at the maxilla and mandible in a single image. Periapical radiographs show teeth in one area of the mouth from crown to root surrounded by alveolar bone and can capture any abnormalities in the tooth and surrounding bone areas. On the contrary, bitewing images are usually used to diagnose caries and assess the bone level, with only a proportion of tooth and bone shown on the image.

A panoramic radiograph provides a quick overview and diagnosis, but it is insufficient to diagnose initial and minor abnormalities [8]. The distortion and a lack of details prevent accurate and precise bone level measurement as well as identification of bony defects to diagnose periodontitis [9–11] and early caries [12]. Periapical radiographs are usually the primary examination method to diagnose dental and oral abnormalities [13] since they can capture accurate and detailed anatomical structures and are available in almost all dental clinics.

Deep learning (DL) models have been utilized in several medical image analyses to identify abnormalities such as brain tumor segmentation [14], breast cancer diagnosis [15], lung cancer [16], prostate cancer [17], and Parkinson’s disease [18] achieving higher performance than other state-of-the-art methods. In the last few years, DL models have been developed in dentistry to diagnose diseases from dental radiographs, including caries [4], radiographic bone loss (RBL) [3, 19, 20], and periapical lesions [21]. However, although these models have good performance in detecting abnormalities, they cannot recognize tooth numbers to provide detailed diagnostic information on individual teeth, therefore limiting the clinical application of these DL models. Recently, some DL models were developed to segment teeth [22], detect and assign tooth numbers on dental radiographs [23–27], diagnostic charting [28], or detect and classify each tooth into molar, premolar, canines, and incisors [29, 30]. However, all of them were only applicable to panoramic radiographs [22–30]. Furthermore, some studies have been conducted on detecting teeth and assigning tooth numbers in periapical radiographs [6, 31, 32] and bitewing radiographs [33, 34].

Since it is very common that a patient may have different types of dental radiographs, it is essential to develop a DL model able to recognize tooth numbers in multiple types of radiographic images. Furthermore, the tooth numbering model needs to be compatible with other disease diagnostic models to prove its reliability, usability, and applicability in clinical settings. Therefore, our study aimed to develop a model to identify tooth numbers in panoramic and intraoral radiographs and arrange full mouth series (FMS) radiographs into an FMS arrangement template. The proposed model is robust and could be integrated with other dental diagnosis models, such as the periodontitis and caries detection model, to facilitate clinical examination and diagnosis and improve the clinical practice workflow.

## Methods

### Overview of the proposed framework

This study was conducted following the World Medical Association’s Declaration of Helsinki, the study checklist developed by Schwendicke et al. for artificial intelligence in dental research [35] (Supplementary Table 1), and the guidelines of the Standards for Reporting Diagnostic Accuracy (STARD) [36] (Supplementary Table 2). This study was approved by the University of Texas Health Science Center at Houston Committee for the Protection of Human Subjects (HSC-DB-20-1340).

The framework used a matching strategy to match the extracted tooth with the tooth repository or patient’s panoramic radiograph and assigned the tooth number without prior domain knowledge or rule-based information. The method was divided into two parts: (A) Assign tooth number for each tooth in periapical and bitewing radiographs and (B) Arrange the set of radiographs (periapical and bitewing) into an FMS arrangement template. First, image segmentation models were utilized to detect teeth bounding boxes from periapical and bitewing images and extract individual teeth using those bounding boxes. Then, each tooth was matched with the patient’s panoramic radiograph (if available) or tooth repository (if the patient’s panoramic radiograph was unavailable) to assign the tooth numbers. Finally, we repeated this process to all FMS radiographs to arrange them into an FMS arrangement template based on position and tooth numbers.

**Fig. 1:**
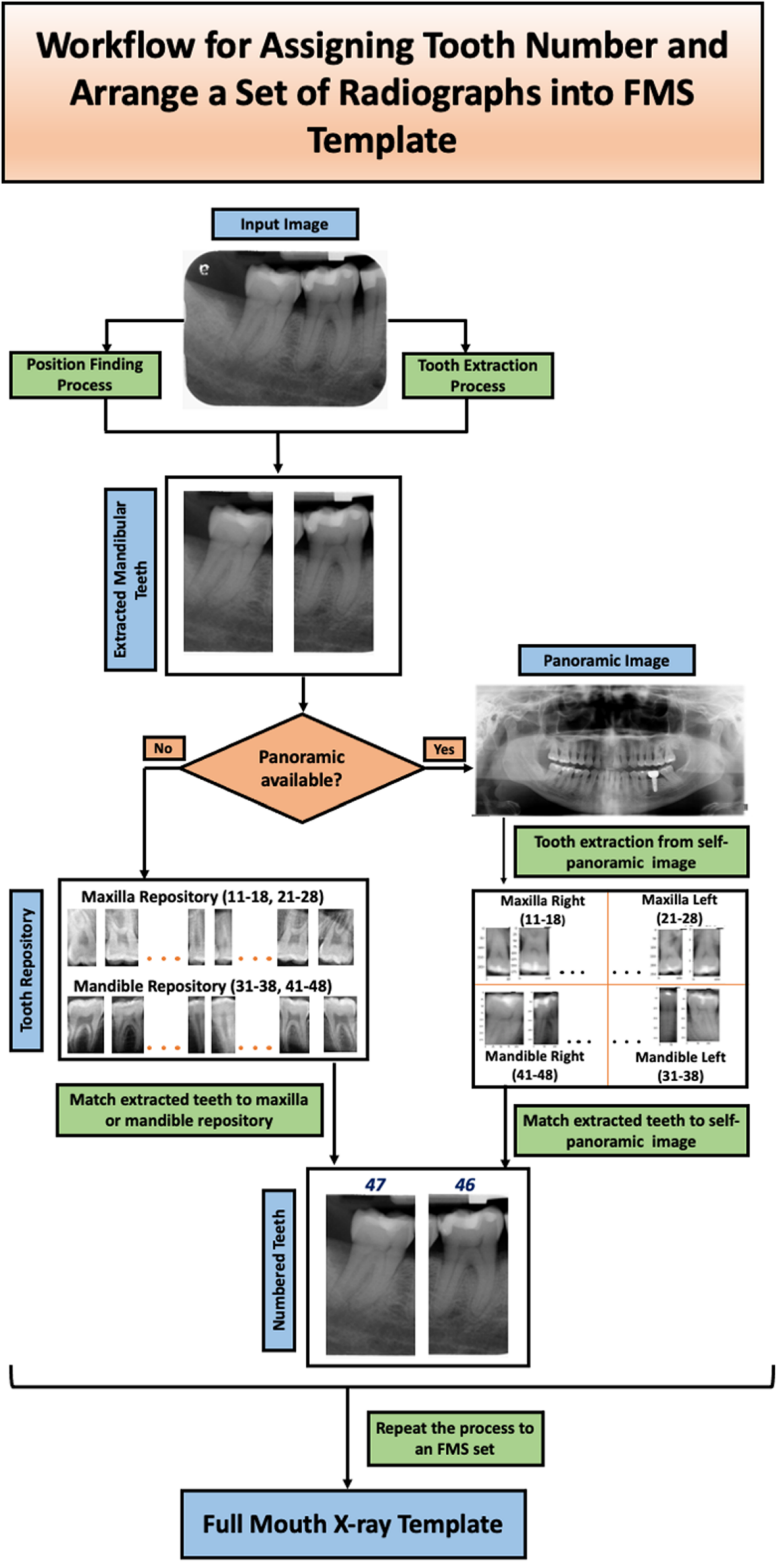
Workflow of the proposed system. Tooth number assignment workflow following the Federation Dentaire Internationale (FDI) tooth numbering system and arranging a set of radiographs into an FMS arrangement template

Figure 1 illustrates the workflow of the proposed system. First, the input image was processed to find the individual tooth’s position and extract it. In this step, the segmentation networks were utilized to generate masks of teeth, bone area, and the cementoenamel junction (CEJ) line from periapical or bitewing radiographs. Tooth and CEJ line masks were required to extract individual teeth from the radiographs. Bone area and CEJ line masks were essential to determine the radiograph’s position (maxilla, mandible, or both maxilla and mandible for bitewing). Then, postprocessing and image analysis steps were implemented to improve the predicted mask quality and extract individual teeth and determine their position from these masks. If the patient’s panoramic radiograph was available, then we also used segmentation network and image analysis methods to obtain all teeth from panoramic images. Next, the extracted teeth from the periapical and bitewing images were matched with either the extracted tooth from the patient’s panoramic image or tooth repository and tooth numbers were assigned using the majority voting of the top 10 matched scores. Finally, we repeated the entire process to a set of radiographs to arrange them into the FMS arrangement template based on their positions and tooth numbers: top layer- maxillary, middle layer- bitewing, and lower layer- mandibular.

### Data, data protection, sampling, sample size, and data processing

Panoramic images from Abdi et al. [37] were used to train the panoramic segmentation model. The link for the dataset is- https://data.mendeley.com/datasets/hxt48yk462/1. This public repository contained 116 panoramic images, and all teeth in those images were annotated by three experts, as mentioned in Abdi et al. [37]. Additionally, 682 periapical and bitewing radiographs were obtained from the private database of UTHealth School of Dentistry (UTSD) and annotated by three experts (two board-certified periodontists and one resident in the periodontics program).

The experts were well-calibrated before annotation. Before starting the annotations, the Dice Similarity Coefficient among the annotators was 0.92 for bone area segmentation and 0.84 for tooth segmentation using three sets of FMS (periapical and bitewing) radiographs. These calibrated experts annotated Region of Interests (ROIs) on images in a secure online platform, Computer Vision Annotation Tool (CVAT). The Digital Imaging and Communications in Medicine (DICOM) files were extracted from electronic health records (EHRs), converted to Portable Network Graphics (PNG) files, and then uploaded to the annotation platform. Multiple ROIs, including tooth, bone area, and caries, were annotated using a polygon, and CEJ was annotated using a polyline. If there was a disagreement for periodontal staging among annotators, majority voting (at least two of the three) was applied for the final stage assignment. For the panoramic image segmentation, we used the gold standard from the repository of Abdi et al. [37]. Three expert dentists manually segmented those images. If there was a conflict during manual segmentation, majority voting was utilized to generate a reliable unified segmentation [37].

All intraoral and panoramic images were randomly extracted from EHRs of patients (age ≥ 18) diagnosed with periodontitis, gingivitis, and/or caries in UTSD. In our dataset, the prevalence of periodontitis at the tooth level was 19.5% for stage 1, 12% for stage 2, and 12.7% for stage 3. The prevalence of caries was 26% at the tooth level. Images with no teeth, implants only, or severe teeth crowding were excluded. All digital intraoral images were taken using the KaVo™ FOCUS™ (KaVO Dental, Bieberich, Germany) wall-mounted x-ray unit with the standard Rinn XCP-ORA PSP holder system. The panoramic images were taken with the Planmeca Promax S3 Panorex + Ceph - Dimax 3 (Planmeca, Helsinki, Finland) with the head position held in the Frankfort horizontal plane. All images were reviewed and approved by the radiology technicians or radiologists at UTSD. The three experts also reviewed these images to ensure image quality before annotations.

The panoramic images were used to train a segmentation model to spot individual teeth. The periapical and bitewing radiographs were used to train bone area, tooth, and CEJ line segmentation models. The dataset was randomly divided into 80% for training and 20% for internal validation. In addition, 1240 periapical and bitewing radiographs from 62 patients (obtained from the private database of UTSD) different from the training and internal validation cohort were utilized for testing to avoid data snooping.

All panoramic, periapical, and bitewing images were resized to 512 × 512. In addition, the periapical and bitewing images were converted to heatmap images before the model training because heatmap images could provide better visual cues for models leading to better feature extraction due to the variation of color intensity [15, 38].

### Model, model parameters, and training

**Fig. 2:**
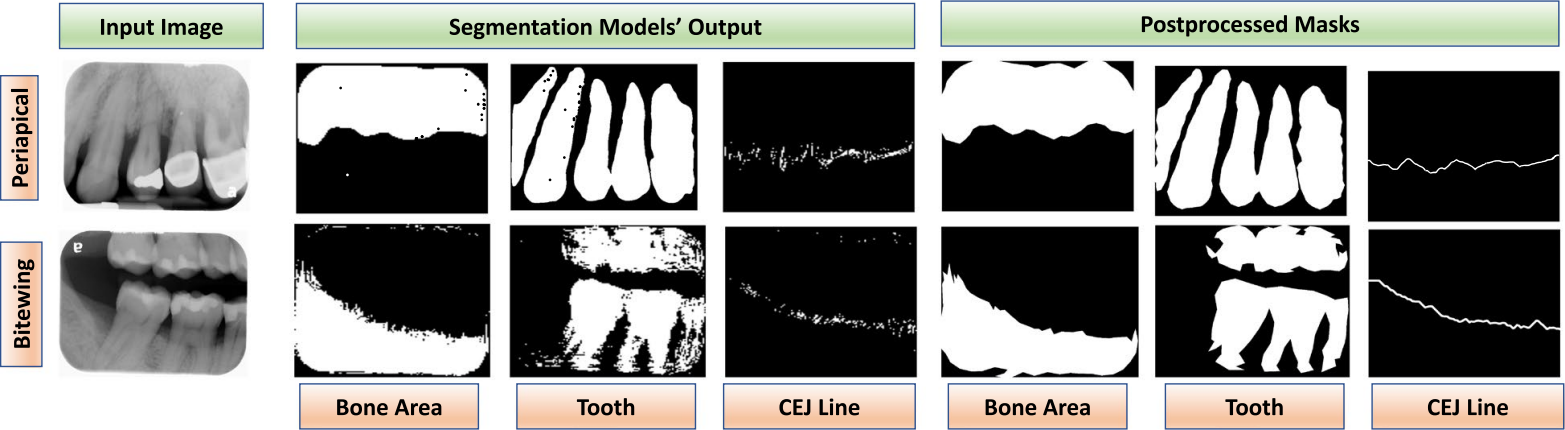
Segmentation models’ output. Segmentation models’ outputs and post-processed masks of bone area, tooth, and the CEJ line. Image postprocessing steps are utilized to improve the quality of the model-generated masks

U-Net [39] segmentation model was used to segment teeth from panoramic images and CEJ lines from periapical images. U-Net with ResNet-34 [40] model was utilized for the bone area and tooth segmentation from periapical and bitewing images. Binary cross-entropy loss was used as a loss function for all segmentation models, and stochastic gradient descent with Adam optimizer was utilized to optimize the model parameters. Additionally, several hyperparameters, such as the number of convolutional layers, the number of kernels in each layer, and kernel sizes, were varied to find the best models. Furthermore, different postprocessing techniques such as Gaussian filtering (remove noises from masks), contour detection (for the bone area and tooth detection) and sliding window to draw connected lines (to provide connected CEJ line) were employed to improve mask quality (Fig. 2).

### Tooth repository using panoramic images

**Fig. 3:**
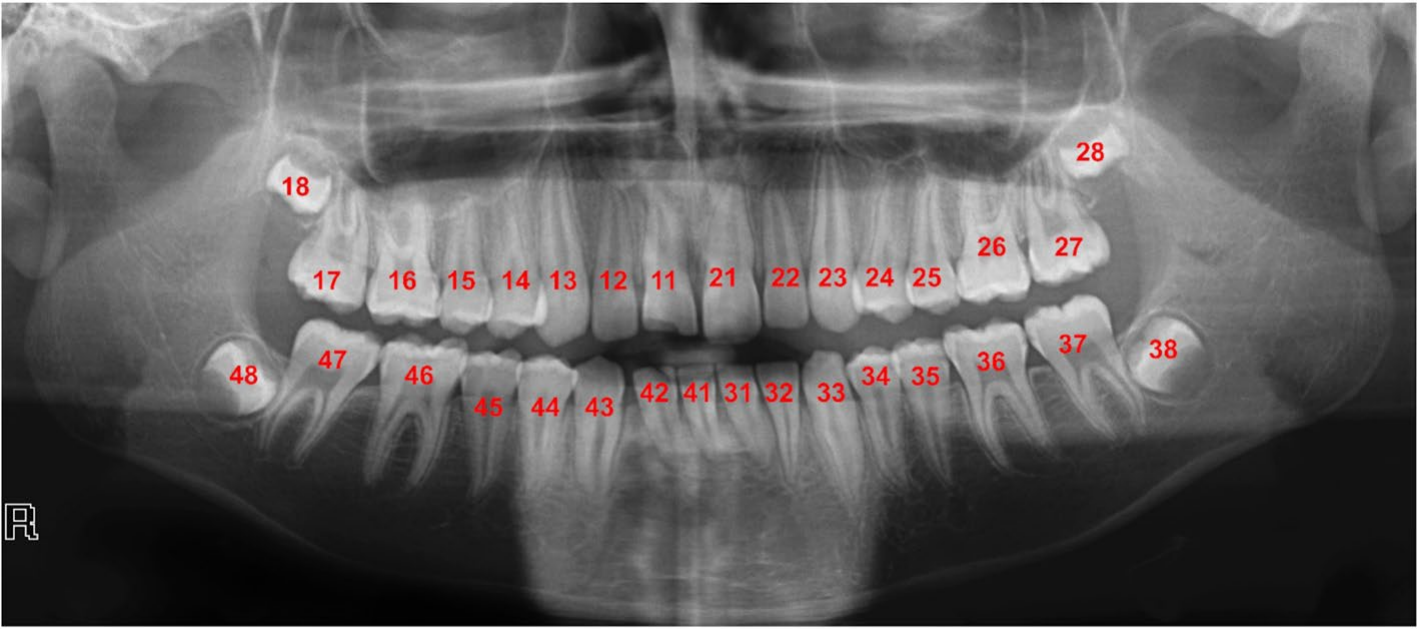
FDI tooth numbering system. A sample tooth repository following the FDI tooth numbering system

Following the Federation Dentaire Internationale (FDI) tooth numbering system [41], a sample tooth repository was prepared as the reference and used to assign numbers to each tooth. This framework used tooth numbers 11–18 (right maxilla), 21–28 (left maxilla), 31–38 (left mandible), and 41–48 (right mandible). Our current repository contains 2094 individual teeth and corresponding tooth numbers from 70 panoramic images (52 from Abdi et al. [37] and 18 from UTSD database) to address different shapes and missing, broken, or irregularly shaped tooths among different people. Figure 3 illustrates a panoramic reference image with the FDI tooth numbering system from the tooth repository.

### Teeth numbering

**Fig. 4:**
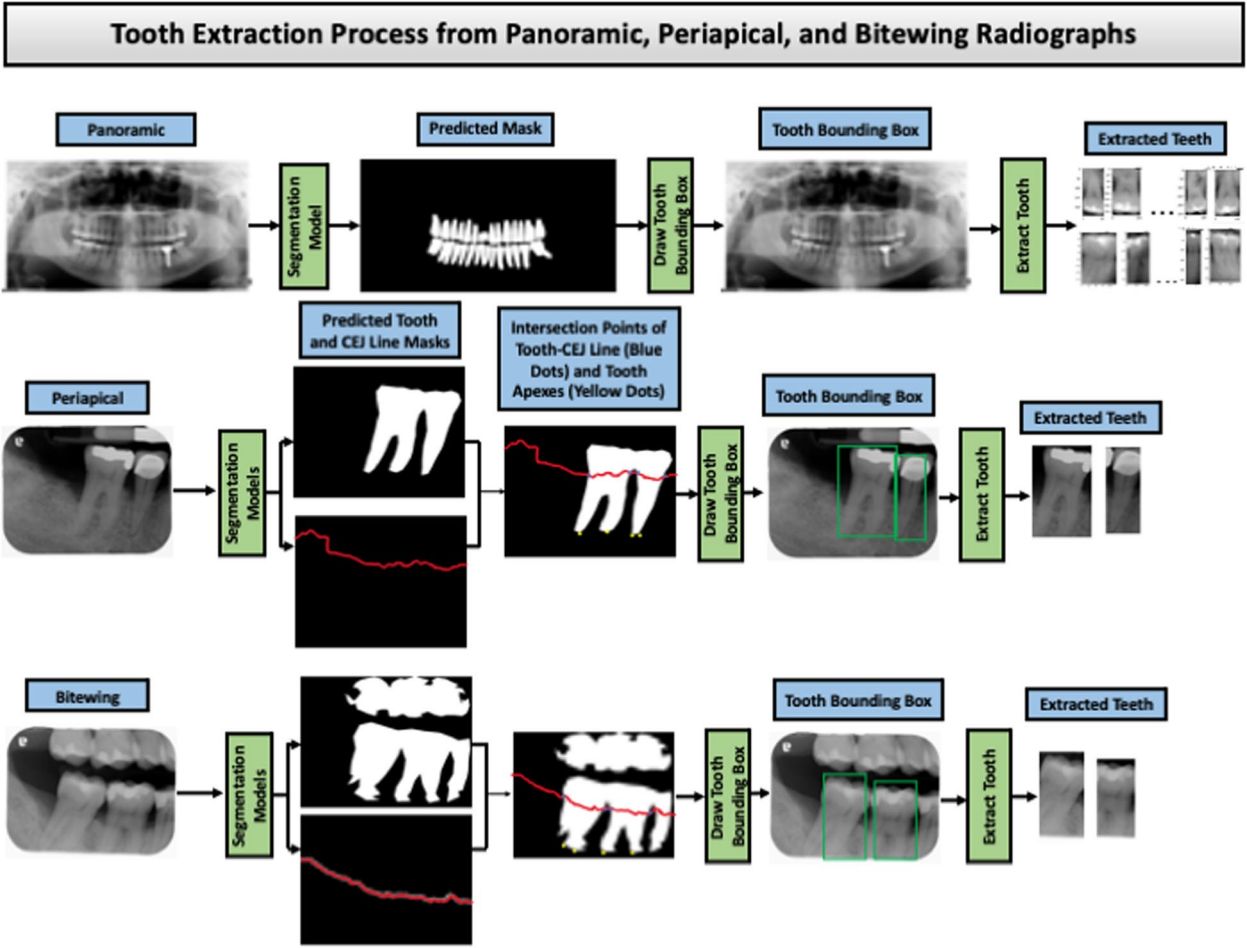
Tooth extraction process. Tooth extraction process from panoramic, periapical, and bitewing radiographs using tooth and CEJ line masks. The bounding box is utilized to extract individual teeth using the intersection points of the tooth-CEJ line and the tooth roots

We extracted individual teeth by detecting the bounding box of each tooth from the predicted masks in periapical, bitewing, and panoramic radiographs. For the panoramic images, a contour detection algorithm [42] was applied to the masks to detect the contours of the teeth. A closed curve or contour was identified as having the same color or intensity in masks using the surrounding relations among the borders of a binary image. These detected contours were used to draw the bounding boxes. For the periapical and bitewing images, the first intersection points of the tooth-CEJ line and root apexes (left and right) were detected for each tooth, and then those four points were used to draw the tooth-bounding box. The individual tooth could be extracted from the panoramic, periapical, and bitewing radiographs (Fig. 4).

1. Tooth Matching via Panoramic View: If the patient had panoramic, periapical, and bitewing radiographs available, then extracted teeth from the periapical and bitewing radiographs were matched with the patient’s panoramic radiograph for assigning tooth number.
2. Repository Matching: If only periapical and bitewing radiographs were available, the extracted teeth were matched to teeth from the repository.

As the extracted teeth from the intraoral radiographs and teeth in the repository were of different sizes, we used a multi-scale matching process where we could vary the extracted teeth size to find the best match. The steps to find the best match are explained in detail in the Multi-scale Matching Process section in the Supplementary material.

### FMS arrangement

**Fig. 5:**
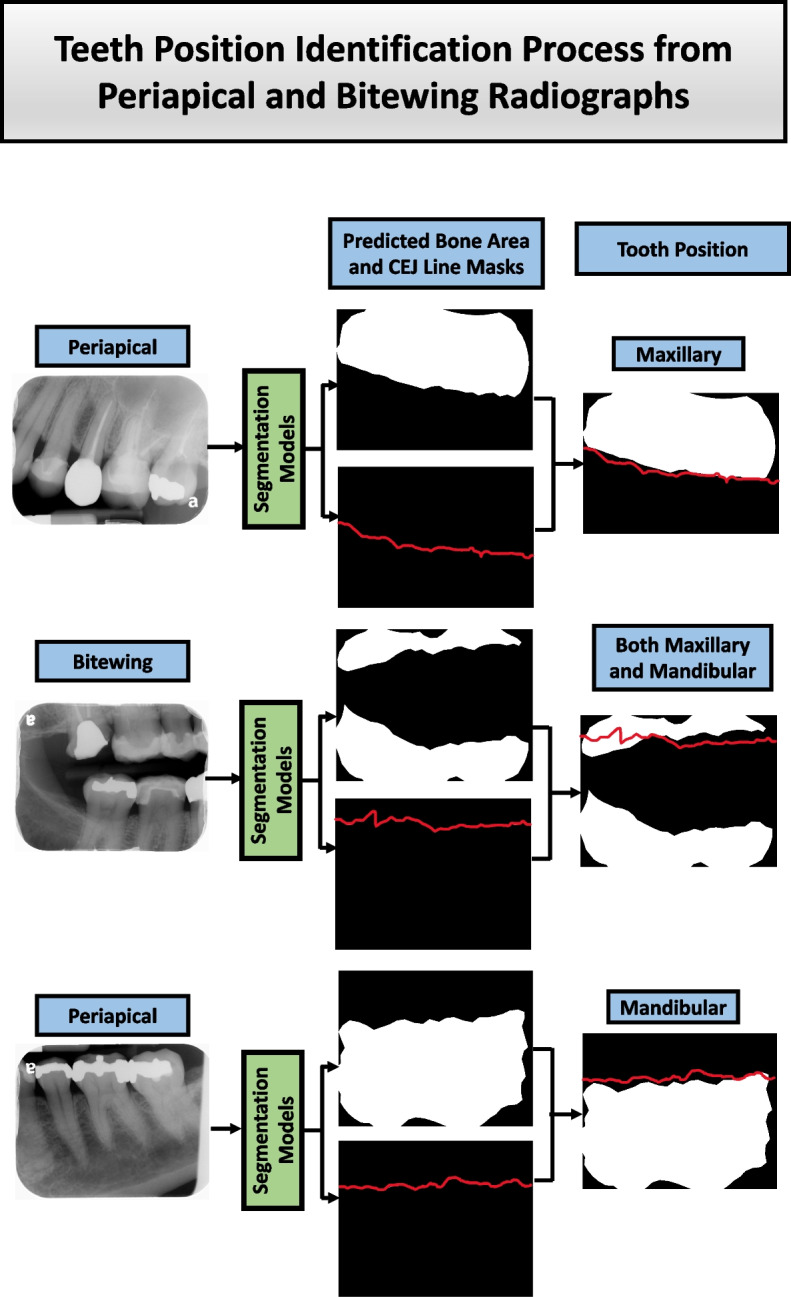
Position determination process. Tooth position identification process using bone area and CEJ line masks for periapical and bitewing radiographs. If the bone area resides above the CEJ line, then maxillary; if there are two sets of the bone area, then bitewing radiographs, and if the bone area resides below the CEJ line, then mandibular radiographs

**Fig. 6:**
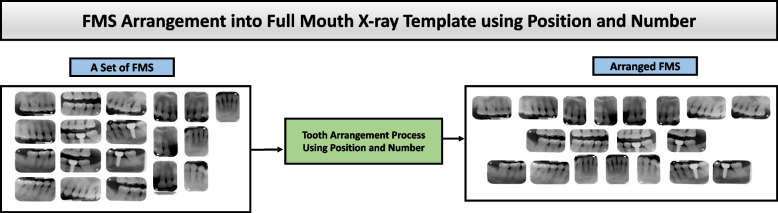
FMS arrangement. A set of periapical and bitewing radiographs arrangement into FMS arrangement template. The top row is maxillary radiographs, the middle row is bitewing radiographs, and the bottom row is mandibular radiographs

The steps to arrange the periapical and bitewing radiographs into the FMS template are given below:

A Identify maxillary, mandibular, and bitewing radiographs using bone area and CEJ line masks (Fig. 5).
  a If there are two bone areas or two sets of teeth, then bitewing.
  b If the bone area is above the CEJ line on the image, then maxillary.
  c If the bone area is below the CEJ line on the image, then the mandibular.
B Extract each tooth using the tooth and CEJ masks.
C Get the tooth number for periapical and bitewing images using the process explained in the section “Tooth Numbering.”
D Arrange each radiograph based on its position and tooth number (Fig. 6).

### Performance metrics

The performance of the image segmentation models was evaluated using Dice Similarity Coefficient (DSC) and Jaccard Index (JI). DSC (Eq. 1) compares the similarity between the model’s predictions and gold standard. JI (Eq. 2) is used to calculate the intersection between the model segmentation and gold standard regions over their union.

1 $$DSC=\frac{2\times Area\ of\ overlap}{Total\ number\ of\ pixels\ in\ both\ images}$$

2 $$JI=\frac{Area\ of\ overlap}{Area\ of\ union}$$

The performance of the image classification model was evaluated using confusion matrix, sensitivity (Eq. 3), and specificity (Eq. 4).

3 $$Sensitivity=\frac{Number\ of\ True\ Positives}{Number\ of\ True\ Positives+ Number\ of\ False\ Positives}$$

4 $$Specificity=\frac{Number\ of\ True\ Negatives}{Number\ of\ True\ Negatives+ Number\ of\ False\ Positives}$$

We compared the detected bounding box with the gold standard bounding box using JI. If the JI was over 0.7, we considered the bounding box as a successful match. Finally, we evaluated the performance of the proposed tooth numbering system using the following matrices.

5 $$Detection\ Precision=\frac{N_{successful\ match}}{N_{detected\ bounding\ box}}$$

6 $$Detection\ Recall=\frac{N_{successful\ match}}{N_{gold\ standard\ bounding\ box}}$$

7 $$Numbering\ Precision=\frac{N_{true\ positive\ numbering}}{N_{detected\ bounding\ box}}$$

8 $$Numbering\ Recall=\frac{N_{true\ positive\ numbering}}{N_{gold\ standard\ bounding\ box}}$$

Here, *N*_*successful match*_ is the number of successfully matched bounding boxes with JI over 70%, *N*_*detected bounding box*_ is the number of detected bounding boxes, *N*_*gold standard bounding box*_ is the number of gold standard bounding boxes, *N*_*true positive numbering*_ is the matched tooth number.

Tooth numbering accuracy was calculated by the following equation (Eq. 9)

9 $$Tooth\ Number ing\ Accuracy=\frac{Total\ Number\ of\ Correctly\ Numbered\ Teeth}{Total\ Number\ of\ Teeth}$$

The FMS arrangement accuracy was calculated using position accuracy (Eq. 10).

10 $$Position\ Accuracy=\frac{Total\ Number\ of\ Correctly\ Positioned\ Radiographs}{Total\ Number\ of\ Radiographs}$$

### Teeth profiling for dental disorders

The proposed model is self-contained and can be integrated with various dental diagnostic models. We integrated it with different abnormalities detection models, such as periodontal bone loss and caries, to assess the practicality of this framework which facilitates the generation of a comprehensive clinical report with the clinical diagnosis of individual teeth. One hundred fifty periapical images were uploaded to the periodontitis diagnosis report interface, and 50 periapical images with at least one caries lesion were uploaded to the caries detection report interface to assess the accuracy of the tooth number assignment in these interfaces. The following describes a brief overview of the periodontal diagnosis and caries detection models.

#### Periodontal diagnosis

Here, we use the previously developed DL model which integrates the segmentation and classification models and image analysis methods to measure RBL percentage and assign periodontal stages using periapical radiographs [3, 20]. The segmentation models generate the bone area, tooth, and CEJ line masks used to extract individual teeth. Then, all extracted teeth, corresponding bone area, and CEJ line are passed through the classification model to obtain periodontal stages. Besides the classification model, we use image analysis and rule-based methods to calculate the RBL percentage (Eq. 11).

11 $$Radiographic\ Bone\ Loss\ (RBL)=\frac{Length\ from\ CEJ\ Line\ to\ alveolar\ bone\ level}{\ Length\ from\ CEJ\ Line\ to\ root}\times 100$$

Assigning stages using the bone loss percentage is based on the 2018 periodontitis classification [43].

- Stage 1: RBL < 15% (in the coronal third of the root)
- Stage 2: 15% ≤ RBL ≤33% (in the coronal third of the root)
- Stage 3: RBL > 33% (extending to the middle third of root and beyond)

#### Caries detection

A convolutional neural network-based segmentation model, U-Net with attention [44], was trained to detect caries. U-Net utilized a shortcut path to combine the spatial features of the encoding path with the decoding path. In addition, soft attention was used to suppress irrelevant spatial information and reduce the transfer of redundant features. After model prediction, several postprocessing methods were utilized to remove the noise from the masks and identify the contour of caries on each tooth in the periapical and bitewing images.

### Code and data availability

All codes and data necessary to reproduce the results are available at https://github.com/tanjidakabir/TK_Tooth_Number_Code

## Results

### Segmentation task

DSC and JI were used to evaluate the segmentation models’ performance. We have run the segmentation models’ multiple times on different seeds to find the mean and standard deviation of dice similarity score and Jaccard Index on test data, as reported in Table 1. The average DSC score of the segmentation models for panoramic and periapical radiographs is over 0.88.

**Table 1:** Dice Similarity Coefficient (DSC) and Jaccard Index (JI) for segmentation models

Image Type	Number of Cases	Number of Images	Segmented Area	DSC (mean ± std)	JI (mean ± std)
Panoramic	70	70	Tooth	0.94 ± 0.0115	0.88 ± 0.0058
Periapical	46	644	Bone Area	0.96 ± 0.0007	0.93 ± 0.0012
	46	644	Tooth	0.93 ± 0.0295	0.88 ± 0.0499
	46	644	CEJ Line	0.91 ± 0.0454	0.88 ± 0.0175

### Periodontal diagnosis and caries detection task

Tables 2 and 3 provide the confusion matrix, sensitivity, specificity, and the area under the receiver operating curve (AUC-ROC) values of the periodontal model for assigning periodontal stages. The periodontal model was tested on 55 periapical radiographs from 10 individual cases.

**Table 2:** The confusion matrix of periodontal diagnosis model for assigning periodontitis stages

True Stage	Predicted Stage		
	Stage 1	Stage 2	Stage 3
**Stage 1**	73	1	0
**Stage 2**	5	29	10
**Stage 3**	1	5	42

**Table 3:** The sensitivity, specificity, and AUC-ROC of the periodontal diagnostic model for different stages

	Sensitivity	Specificity	AUC-ROC
**Stage 1**	0.99	0.93	0.89
**Stage 2**	0.66	0.95	0.90
**Stage 3**	0.88	0.92	0.90

DSC and JI were applied to evaluate the caries detection model performance. A total of 32 periapical and bitewing radiographs from 7 patients were selected to test the caries detection model. The average DSC and JI for the caries detection model are 0.88 ± 0.0032 and 0.80 ± 0.0055.

### Tooth numbering task

Table 4 demonstrates the precision and recall for detecting and assigning tooth numbers of the proposed tooth numbering system. We observed that tooth matching via the patient’s panoramic image performs better than repository matching and outperforms the other state-of-the-art models. We also compared the performance of the proposed tooth matching system using patients’ panoramic images and repository matching with previous work (Table 4). Additionally, the accuracy of tooth numbering was still high in the images with missing teeth and/or restorations. In the 26 periapical images with missing teeth, the accuracy was 94%. In the 48 periapical radiographs with restorations, the accuracy was 89%. Here, the bold numbers indicate the best performance score of the proposed system.

**Table 4:** Performance evaluation of the proposed tooth numbering system

	Test Images	Bounding Box Exists	Bounding Box Matched	Detection Precision	Detection Recall	Numbering Precision	Numbering Recall
Tooth matching via panoramic view	240	745	743	**0.99**	**0.99**	**0.96**	**0.96**
Repository matching	1240	3059	3049	0.99	0.99	0.87	0.87
Chen et al. [6]	250	871	868	0.98	0.98	0.91	0.91
Zhang et al. [31]	200	–	–	–		0.95	0.96
Görürgoz et al. [32]	156	–	–	–		0.78	0.98

### FMS arrangement

Our proposed FMS arrangement task based on the segmentation and repository matching process demonstrated average accuracy of 92.5% for arranging periapical and bitewing radiographs on the FMS arrangement template. We tested the arrangement task on 30 cases, where each case had 14 periapical images (seven maxillary and seven mandibular) and four bitewing images. The position accuracy for the maxillary and mandibular periapical arrangement was 95%, and the bitewing arrangement was 90%. However, the accuracy for bitewing images was decreased due to the partial visibility of molar and premolar teeth.

### Clinical report generation

**Fig. 7:**
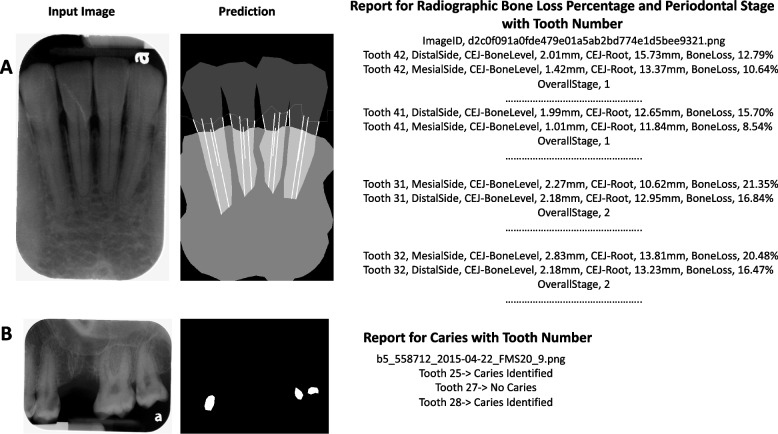
Report of tooth number for different dental abnormalities. A sample report for (**A**) radiographic bone loss (RBL) percentage with periodontal stage and (**B**) caries with tooth number in periapical radiographs. The first panel refers to the input image. The second panel is the model’s prediction. The third panel is the system-generated clinical report for different dental abnormalities with tooth numbers

The proposed interfaces demonstrate the integration of the tooth numbering framework and disease diagnostic models. Figure 7A illustrates a sample of the clinical report for RBL percentage and periodontal stage assignment with tooth numbers using the rule-based method and classification networks. Figure 7B shows a clinical report for caries with the corresponding tooth numbers. The accuracy of tooth numbering was 93% in the 200 tested radiographs.

## Discussion

Using artificial intelligence to recognize tooth numbers from radiographs to facilitate clinical applications is essential. This study demonstrated the high performance of tooth number recognition from both intraoral and panoramic radiographs. Previous studies, which investigate tooth numbering using DL models, are mostly focused on panoramic images.

To the best of our knowledge, few studies have been conducted on detecting teeth and assigning tooth numbers in periapical radiographs. Zhang et al. [31] designed a cascaded CNN model to assign tooth numbers in periapical radiographs, achieving 95.8% precision and 96.1% recall. Besides the CNN model, they needed additional rule-based information to check the proper tooth sequence. Görürgoz and his team [32] developed a Faster Region-based CNN(R-CNN) algorithm for detecting and numbering teeth on periapical images, but their precision and sensitivity are 0.7812 and 0.9867. Chen et al. [6] used faster R-CNN to detect and number teeth in periapical radiographs, achieving 91% precision and recall. However, that model needed to impose prior domain knowledge and rule-fitting to improve detection and numbering precision. Yasa et al. [33] developed a faster R-CNN model for identifying and numbering teeth in bitewing images, but the exact boundary of the teeth could not be recognized. Yaren et al. [34] developed a Mask R-CNN to assign tooth numbers only in bitewing images but did not show their integrity with other dental diagnosis models such as caries or restorations.

Our proposed tooth numbering framework outperformed previous works, achieving higher precision and recall without prior domain knowledge or rule-fitting techniques. We employed majority voting to find the best match and remove biases because people may have different tooth shapes due to some abnormalities. Furthermore, the proposed framework could assign tooth numbers even with the presence of a missing tooth as the matching process relies on either patient’s panoramic radiographs or tooth repository.

The proposed framework is able to assign tooth numbers in both periapical and bitewing images with a minimum error rate. It can also arrange a set of FMS images in a template in the correct order. Additionally, the tooth numbering model could be easily integrated with other dental diagnosis models to generate clinical reports, which can be used to assist clinicians in making accurate diagnoses and validating clinical chartings.

This proposed tooth numbering model is primarily used to improve the comprehensiveness of deep learning-based diagnostic tools based on radiographic images. Potentially, the findings of radiographic images and clinical chartings (e.g., periodontal charting) can be mutually validated. For example, if severe bone loss is detected at the mesial site of tooth #30 on the radiograph by the deep learning models, but the pocket depth at the mesial site of tooth #30 on the periodontal charting is shallow, it is possible that the periodontal charting is inaccurate. Clinicians can make necessary changes based on the findings from the radiographic images and chartings. Finally, it is planned to integrate diagnostic and tooth numbering models with the EHR system to have clinical chartings validated by deep learning-based clinical reports for future clinical applications. This integration will improve the accuracy of clinical diagnosis and streamline the clinical workflow. However, the integration process can be challenging due to incompatible software and difficulty in designing user-friendly interfaces.

In addition, the proposed framework required significantly less (30x) processing time in comparison to examiners to assign tooth numbers with dental diagnosis. For example, the average time for tooth number assignment with the periodontal diagnosis was 7 seconds for each radiograph while the examiners required in average 218 seconds to complete the radiographic bone loss measurement and enter it into the system.

However, our present study has some limitations. First, the current framework cannot handle incorrectly oriented intraoral radiographs such as upside-down or mirrored. Improvement is needed to identify the anatomical landmarks, such as maxillary sinus and mental foramen to reorientate and assign the toot number for those images. Second, the accuracy of bitewing images can be improved by integrating the explementary bitewing images into the repository. Third, the proposed framework cannot assign the correct tooth number for full-arch dental implants and broken or irregular-shaped teeth without a panoramic radiograph. We will integrate the dental implant recognition framework into the proposed system to generate a complete framework of the tooth recognition system. Finally, the FDI tooth numbering system is used for assigning tooth numbers in the current model because it is the most common numbering system worldwide. This model can be easily modified for applications in the United States to assign tooth numbers based on the universal tooth numbering system.

## Conclusions

The proposed DL framework focuses on assigning tooth numbers on intraoral radiographs and provides high-throughput diagnostic assistance in clinical settings. This framework works on a single intraoral radiograph and combines a set of periapical and bitewing radiographs to arrange them into the FMS template based on their positions. Besides, the tooth numbering model can be integrated into other dental abnormality detection systems to assist dentists in creating an automated and time-efficient treatment plan.

## Supplementary Information

**Additional file 1: Supplementary Table 1.** Items to be considered when planning, conducting, and reporting AI studies in dental research. **Supplementary Table 2.** Items to be considered when reporting diagnostic accuracy.

## Data Availability

The datasets used and/or analyzed during the current study are available from the corresponding author upon reasonable request. The sample code and data for reproducibility is available at the following link: https://github.com/tanjidakabir/TK_Tooth_Number_Code.

## Abbreviations

AI: Artificial Intelligence
CBCT: Cone-Beam Computed Tomography
CEJ: Cementoenamel Junction
CNN: Convolutional Neural Network
DL: Deep Learning
DSC: Dice Similarity Coefficient
FDI: Federation Dentaire Internationale
FMS: Full Mouth Series
JI: Jaccard Index
RBL: Radiographic Bone Loss
R-CNN: Region-based Convolutional Neural Network
ResNet: Residual Network
