# Supplementary material for: A comprehensive artificial intelligence framework for dental diagnosis and charting
Source: BMC Oral Health. 2022 Nov 9;22:480. doi: 10.1186/s12903-022-02514-6 (PMC9647924; doi:10.1186/s12903-022-02514-6)
Supplement: Supplementary file 1 — Additional file 1: Supplementary Table 1. Items to be considered when planning, conducting, and reporting AI studies in dental research. Supplementary Table 2. Items to be considered when reporting diagnostic accuracy. [file 12903_2022_2514_MOESM1_ESM.docx]

**Supplementary**

Schwendicke F, Singh T, Lee JH, Gaudin R, Chaurasia A, Wiegand T, Uribe S, Krois J; IADR e-oral health network and the ITU WHO focus group AI for Health. Artificial intelligence in dental research: Checklist for authors, reviewe(1)rs, readers*. J Dent*. 2021 Apr;107:103610. doi: 10.1016/j.jdent.2021.103610. Epub 2021 Feb 22. PMID: 33631303.

**Supplementary Table 1. Items to be considered when planning, conducting, and reporting AI studies in dental research.**

| Item  No | Planning and Conducting |  | Reported on page No. |
| --- | --- | --- | --- |
| 1. | Study Goal | Consider relevance, scope and  meaning and limitations of the AI  application. | 3-5, 18-21 |
| 2. | Study Focus | Clarify if developing a new or  validating an existing model, or if  scope diagnostics or prognostics etc.. | 3-5, 18-21 |
| 3. | Data | Scrutinize the available dataset and  mitigate bias, ensuring  generalizability | 7-9 |
| 4. | Study Aim | Define if the study is exploratory or  hypothesis-testing and consider  implications for study conception. | 3-5 |
| 5. | Reference Test | Decide on a justifiable basis for a  method to establish a reference test,  especially when involving multiple  annotators. | 7,8 |
| 6. | Clustering | Consider clustering of teeth and patients in your dataset, for example during data partitioning ("data snooping bias"). | 7-9 |
| 7. | Test Dataset | Report test metrics from an independent test dataset. | 11-13 |
| 8. | Computational Resources | Consider resources when working with larger datasets or complex models. | NA |
| 9. | Comparators  Reporting | Compare your model against relevant comparators (experts, other models) using meaningful metrics. | 18,19 |
| 10. | Title | Define that any kind of AI was used, specify which one and for which focus and problem. | 3, 4 |
| 11. | Abstract | Present a structured summary of the study's aim, methods, results, and conclusion. | 1, 2, 3 |
| 12. | Introduction | Sum up the clinical background and need of AI solution; achievements and limitations so far; goal of the study; hypothesis (if needed). | 3-5 |
| 13. | Study design | Assist the reader in understanding your study by providing an overview about the study goal, data characteristics, modeling techniques, evaluation and scope. | 3-7 |
| 14. | Data | Give details towards the source of data for training and testing, in- and exclusion criteria, sampling framework, fit to target population, heterogeneity, partitioning, and if and where it can be accessed (or why not). | 7-9 |
| 14 a. | Sampling | Provide inclusion and exclusion criteria, case definition, image type and quality, data source(s)/centers, sampling strategy and information towards heterogeneity. | 7-9 |
| 14 b. | Data Protection | Provide information how data protection requirements were fulfilled. | 7-9 |
| 14 c. | Missing data | Explain how missing data was handled. | NA |
| 14 d. | Data Processing | Lay out how data processing (extracted, transposed, loaded, preprocessed) was performed. | 7-9 |
| 15. | Reference Test | Explain how the reference test was generated, including case definition, grading schemes, test thresholds and unification strategies for multiple labels. | NA |
| 16. | Sample Size | If your study is hypothesis-testing, provide information how your arrived at your test dataset sample size. | NA |
| 17. | Model | Provide detail information on model inputs, outputs, intermediate layers, pooling, normalization, regularization, and activation, as well as software packages and hardware used. The structure of the model may be presented. | 9 |
| 17 a. | Model parameters | Describe how the model parameters were initialized. | 9 |
| 18 | Training | Describe the training procedures including data augmentation techniques, criteria used for stopping the training, hyperparameters and hyperparameter search strategy. For neural networks, at least the learning rate schedule, optimization method, batch size, dropout rates, regularization parameters (if any) and number of epochs should be provided. | 9 |
| 19 | Justify the best-performing model | Describe the method and model metric to select the final model and evaluate it against the hold-out test set. | 15-18 |
| 20 | Evaluation | Describe the primary outcome and outcome metric. Consider further outcomes with relevance to your question. | 3-5, 15-18 |
| 21 | Uncertainty | Describe how uncertainties in the model results (comparisons, subgroups) are reflected on. | 15-18 |
| 22 | Explainability | Lay out how explainability, trustworthiness, and transparency were assessed. | 7-9 |
| 23 | Results | Provide information on flow of data, including those in- and excluded, and data partitions into training, validation and test dataset. Characterize the dataset. | 7-9, 15-19 |
| 23 a. | Performance Metrics and Data Partitions | The final model's performance on the test partition should be provided in detail and benchmarked against current technical standards. Provide uncertainty estimates. Provide information to understand incorrect predictions and explainability. | 15-19 |
| 24 | Discussion | Provide a summary, a strengths and limitations sections, a section on findings and their implications, and one on future directions. | 18-21 |
| 25 | Other | Provide information towards authorship and registration, study protocol and potential conflicts of interest. | 21,22,25-28 |

Bossuyt PM, Reitsma JB, Bruns DE, Gatsonis CA, Glasziou PP, Irwig L, et al. STARD 2015: an updated list of essential items for reporting diagnostic accuracy studies. BMJ [Internet]. 2015 Oct 28;351:h5527. Available from: http://dx.doi.org/10.1136/bmj.h5527

**Supplementary Table 2. Items to be considered when reporting diagnostic accuracy.**

| Section and topic |  | No | Item | Reported on page No. |
| --- | --- | --- | --- | --- |
| Title or abstract |  | 1 | Identification as a study of diagnostic accuracy using at least one measure of accuracy (such as sensitivity, specificity, predictive values, or AUC) | 1-3 |
| Abstract |  | 2 | Structured summary of study design, methods, results, and conclusions (for specific guidance, see STARD for Abstracts) | 1-3 |
| Introduction |  |  |  |  |
|  |  | 3 | Scientific and clinical background, including the intended use and clinical role of the index test | 3,4 |
|  |  | 4 | Study objectives and hypotheses | 5 |
| Methods |  |  |  |  |
|  | Study design | 5 | Whether data collection was planned before the index test and reference standard were performed (prospective study) or after (retrospective study) | 5,6 |
|  | \| Participants \| \| --- \| | 6 | Eligibility criteria | 7-9 |
|  |  | 7 | On what basis potentially eligible participants were identified (such as symptoms, results from previous tests, inclusion in registry) | 7-9 |
|  |  | 8 | Where and when potentially eligible participants were identified (setting, location, and dates) | 7-9 |
|  |  | 9 | Whether participants formed a consecutive, random, or convenience series | 7-9 |
|  | Test methods | 10a | Index test, in sufficient detail to allow replication | 8,9,11-13 |
|  |  | 10b | Reference standard, in sufficient detail to allow replication | 8,9,11-13 |
|  |  | 11 | Rationale for choosing the reference standard (if alternatives exist) | NA |
|  |  | 12a | Definition of and rationale for test positivity cut-offs or result categories of the index test, distinguishing pre-specified from exploratory | 11-14 |
|  |  | 12b | Definition of and rationale for test positivity cut-offs or result categories of the reference standard, distinguishing pre-specified from exploratory | 11-14 |
|  |  | 13a | Whether clinical information and reference standard results were available to the performers or readers of the index test | 14 |
|  |  | 13b | Whether clinical information and index test results were available to the assessors of the reference standard | 14 |
|  | Analysis | 14 | Methods for estimating or comparing measures of diagnostic accuracy | 11,12 |
|  |  | 15 | How indeterminate index test or reference standard results were handled | NA |
|  |  | 16 | How missing data on the index test and reference standard were handled | NA |
|  |  | 17 | Any analyses of variability in diagnostic accuracy, distinguishing pre-specified from exploratory | NA |
|  |  | 18 | Intended sample size and how it was determined | -9 |
| Results |  |  |  |  |
|  | Participants | 19 | Flow of participants, using a diagram | NA |
|  |  | 20 | Baseline demographic and clinical characteristics of participants | 7-9 |
|  |  | 21a | Distribution of severity of disease in those with the target condition | NA |
|  |  | 21b | Distribution of alternative diagnoses in those without the target condition | NA |
|  |  | 22 | Time interval and any clinical interventions between index test and reference standard | NA |
|  | Test results | 23 | Cross tabulation of the index test results (or their distribution) by the results of the reference standard | NA |
|  |  | 24 | Estimates of diagnostic accuracy and their precision (such as 95% confidence intervals) | NA |
|  |  | 25 | Any adverse events from performing the index test or the reference standard | NA |
| Discussion |  |  |  |  |
|  |  | 26 | Study limitations, including sources of potential bias, statistical uncertainty, and generalisability | 18-21 |
|  |  | 27 | Implications for practice, including the intended use and clinical role of the index test | 18-21 |
| Other information |  |  |  |  |
|  |  | 28 | Registration number and name of registry | 5 |
|  |  | 29 | Where the full study protocol can be accessed | 5 |
|  |  | 30 | Sources of funding and other support; role of funders | 25,26 |

**Multi-scale Matching Process:**

1. Take an image from the repository, slide it over the input image to compare it with every same-sized sub-image from the input image, and get the highest correlation coefficient.
2. Rescale the input image and repeat step 1. After looping over all scales of the input image, keep the record of the largest correlation coefficient.
3. Repeat steps 1 and 2 for all images in the repository and records the ten largest correlation coefficients and corresponding tooth numbers of the images in the repository.
4. Finally, perform majority voting of the top ten matched tooth numbers to assign the final tooth number to the input image.
